# On Employment of Ergot of Rye in Some Forms of Retention of Urine

**Published:** 1853-08

**Authors:** 


### *On Employment of Ergot of Rye in some forms of Retention of Urine.* By M. Passot, of Lyons.

Ergot of rye has not only the property of exciting the uterine contractions in cases of inactivity of the uterus, but is also very efficacious in the retention of urine which is caused by atony and paralysis of the bladder. MM. Baudin and Payan of Aix, were the first who endeavored to demonstrate that this agent does not act on the uterus alone, but rather on the lower part of the spinal cord. They also speak very highly of it as well in the affection which we have now under consideration, as in weakness or paralysis of the lower extremities.

Drs. Kinsley, Canuto-Canuti, Sainmont de Rocroy, Allier of Marcigny, have also recorded cases which bear favorable testimony to the utility of ergot in paralysis of the bladder. I will now briefly mention some of them.

Captain B., aged 60, suffering from dysuria, which had increased greatly during the last three months, until it suddenly changed to a complete retention, which necessitated the employment of the catheter several times a day. For two months a host of remedies were used without avail; there was not the slightest improvement. The prostate became enlarged, and the patient suffered much from the use of the catheter, which had to be passed twice every day.

Fifty centigrammes (about eight grains) of ergot of rye, infused in a cupful of boiling water, was administered three times a day. At the expiration of six hours, the patient passed a small quantity of urine, and required the use of the catheter only once in the day. Afterwards it was only passed once in the forty-eight hours, and after ten days the bladder was left to itself.—(*Kinsley's Journal des Comm. Med. Chir.*, March, 1844.)

A lady, aged about 75, was affected with paralysis of the bladder, which for a long time required the use of the catheter. Ergot was prescribed in doses of fifteen decigrammes, (about twenty-eight grains,) in infusion. On the sixth day of this treatment, it was no longer necessary to pass the instrument, the patient being able to pass water spontaneously.—(*Canuto-Canuti*, *Bull. des Sciences Med. de Bologne*, 1845).

A man named Rosseau, aged 58, of a nervous temperament, was, in consequence of a fit of passion, attacked with a complete inability to urinate. The bladder was obliged to be emptied by the catheter. The inertia of this organ continued in spite of cold injections into it, cold enemata, the application of ice, also a blister to the hypogastrium.

Strychnine applied, by means of ointment, as a dressing to the blister, also by frictions in the axillæ, on the following day produced cramps in the legs and arms, and was presently accompanied by stiffness, so that it became necessary to discontinue the use of this remedy. There was not the slightest action on the bladder. It was at this stage that the author conceived the idea of giving ergot of rye. He prescribed six grammes, (about ninety-two grains,) coarsely powdered, to be put into a litre (about thirty-four ounces) of water, macerate for two days, filtered, and injected cold into the bladder. Seven minutes afterward, the patient experienced a desire to urinate, which, however, he could not then satisfy. The next morning the injection was again administered. Eight minutes after, he had vesical tenesmus, and then spontaneous emission of urine. The injections were continued for some days. The cure was complete.—(*Sainmont Gazette des Hopitaux*, 1848).

In 1848, Dr. Allier, of Marcigny, sent a letter to the National Academy of Medicine, in which he gives as the result of his observations, that in only one out of fourteen cases ergot proved of no use.

I also am in possession of some cases which, in an incontestable manner, prove that ergot is capable of restoring the contractility of the bladder. The following is the most remarkable:—In the month of July, 1846, I was consulted by M. H., aged 60, of a dry constitution and a very well marked nervous temperament. M. H. admits having indulged both in venereal excesses and in the excesses of the table, and it is these that he blames for the vesical paralysis from which he is now suffering, and which requires the catheter twice a day; otherwise there is no symptom of organic alteration, no fever, no enlarged prostate. The canal of the urethra is free, although its entire length and the urine when drawn off is perfectly clear. After having experienced the uselessness of tincture of cantharides and blistering the hypogastrium, I used the following prescription:—

Freshly powdered ergot, . . . . . . . 2 grammes (30 grains).
Mucilage, . . . . . . . . . . . . . . 120 " (31 ounces).
A tablespoonful every half hour (shake the bottle).
Ergot of rye, powdered, . . . . . . 15 decigrammes (23 grains).
Cocoa butter, . . . . . . . . . . . a sufficiency.
To be made into two suppositories; one of them to be introduced night and morning.

On the same day, at the expiration of some hours, M. H. felt a desire to micturate. At my evening visit, I ordered a bath. The patient was scarcely in it before micturition took place spontaneously and with force. From this time to his death, M. H. has passed water freely and without the assistance of the instrument. I should add, that to make certain of the cure, I continued the remedy for three or four days, but in a decreasing dose. M. H. died the 30th of January, 1848, of an acute pleuro-pneumonia, during the course of which not a single morbid symptom appeared in the bladder. It is therefore certain that ergot cures retention of urine which depends on pure and simple atony or paralysis of the bladder. But with regard to paralysis consecutive to apoplexy, or depending on other affections of the nervous centers, it is well known that they are unaffected by the remedy we are treating of.—*Gaz. Med. de Lyon* and *Rev. Med.-Chir. de Paris.*

